# Features Associated With Quality of Life Impairment in Hidradenitis Suppurativa Patients

**DOI:** 10.3389/fmed.2021.676241

**Published:** 2021-04-27

**Authors:** Sylke Schneider-Burrus, Athanasia Tsaousi, Sebastian Barbus, Johannes Huss-Marp, Katrin Witte, Kerstin Wolk, Björn Fritz, Robert Sabat

**Affiliations:** ^1^Interdisciplinary Group of Molecular Immunopathology, Dermatology/Medical Immunology, Charité - Universitätsmedizin Berlin, Berlin, Germany; ^2^Center for Dermatosurgery, Havelklinik, Berlin, Germany; ^3^Psoriasis Research and Treatment Center, Charité - Universitätsmedizin Berlin, Berlin, Germany; ^4^Sanofi-Aventis Deutschland GmbH, Frankfurt, Germany; ^5^Berlin Institute of Health (BIH) Center for Regenerative Therapies, Charité-Universitätsmedizin Berlin, Berlin, Germany; ^6^Department of Dermatology, Venereology, and Allergology, Charité – Universitätsmedizin Berlin, Berlin, Germany; ^7^AbbVie Deutschland GmbH & Co. KG, Wiesbaden, Germany

**Keywords:** skin disease, quality of life, dermatology life quality index, obesity, spondyloarthritis, metabolic syndrome, family history

## Abstract

**Background:** Hidradenitis suppurativa (HS) is a chronic inflammatory skin disease with an adverse impact on patients' quality of life (QoL).

**Objectives:** To quantify QoL impairment in patients in Germany suffering from HS and to identify the parameters associated with QoL impairment.

**Methods:** A non-interventional, cross-sectional, mono-centric study with 500 HS patients. QoL data (measured using the Dermatology Life Quality Index; DLQI) and demographic, anamnestic, clinical, and blood parameters were collected. All patients were examined by dermatologists that documented the skin alterations. QoL data from 462 HS patients were available and evaluated.

**Results:** The mean (± standard deviation) DLQI score of HS patients was 13.18 ± 7.99. Approximately 40% and 20% of HS patients declared very large and extremely large QoL impairment, respectively. The degree of QoL disturbance correlated with the severity of skin alterations, blood leucocyte count and, in particular, with anogenital localization and the presence of nodules and fistulas. Furthermore, QoL impairment was associated with specific comorbidities, such as adiposity and back pain, but not with HS family history. QoL impairment was not influenced by whether or not the patients had undergone resection surgery or antibiotic treatment but was more severe in HS patients that had undergone abscess lancing compared to patients without such treatment in the past.

**Limitations:** It was a mono-centric study and most data were obtained from self-administered patient questionnaires. The association of QoL with type of treatment was analyzed for abscess lancing, resection surgery, and antibiotic treatment. Further therapeutic modalities recommended in the guidelines were not investigated.

**Conclusion:** A profound impairment in QoL was present in patients with HS, and this was higher than that observed in other studied dermatoses. The degree of impairment correlated with the extent of cutaneous and some extra-cutaneous alterations. Surgical and conventional medicamentous therapies of HS were not associated with long-lasting reduction of QoL impairment. Our data support the implementation of patient-reported outcome measures for the assessment of therapy responses.

## Introduction

Hidradenitis suppurativa (HS; also known as acne inversa) is a chronically relapsing inflammatory disease with characteristic alterations in skinfolds (1). HS is estimated to affect around 1% of the general population (2–4), with a common onset in the second and third decade of life (5). The axillary, inguinal, and gluteal areas are most commonly affected. Furthermore, the inner thighs, perineal area, and sub- and infra-mammary skin may be involved (1). HS leads to painful cutaneous lesions with malodorous discharge and causes irreversible destruction of normal skin structure. The clinical manifestation varies and includes recurring inflammatory nodules, abscesses, draining fistulas, and scars (1).

Despite the burden caused by HS alterations, the pathogenetic mechanisms underlying the skin inflammation are still obscure (6). The current model implies that initially, hyperplasia of the follicular epithelium leads to stasis, dilatation, and formation of subcutaneous nodules and propagation of bacteria (6, 7). Later in the process, a rupture of the pilosebaceous unit causes deep dermal abscess formation with purulent exudate. At molecular and cellular level, HS skin lesions are characterized by infiltration of immune cells and strong expression of numerous pro-inflammatory cytokines (8–12). The inflammation leads to the destruction of cutaneous architecture and the development of deep sinus tracts and scarring of the affected skin (6, 13). Furthermore, several inflammatory mediators reach the blood and may contribute to comorbid disorders (14, 15). In fact, HS is frequently associated with both spondyloarthritis and metabolic alterations, which increases the risk of cardiovascular diseases and reduces life expectancy (16–19).

The associated pain, the large amount of purulent secretion, malodor, and disfigurement caused by HS have a profound impact on affected patients, mainly resulting in isolation and fear due to stigmatization in work and personal life (20–25). In 2001, a study by Von der Werth *et al*. with 114 participants indicated that HS causes a reduction in quality of life (QoL) of greater extent than that shown for other skin diseases (20). Additionally, a study with 54 patients by Matusiak *et al*. confirmed these observations and showed that the clinical stage and number of involved areas are relevant factors related to QoL impairment (22). As the awareness and care of patients with HS has changed in recent years, our goal was to characterize the QoL of patients in a large cohort and extend the knowledge regarding parameters associated with a poor QoL.

## Materials and Methods

### Patients

**Figure 1:**
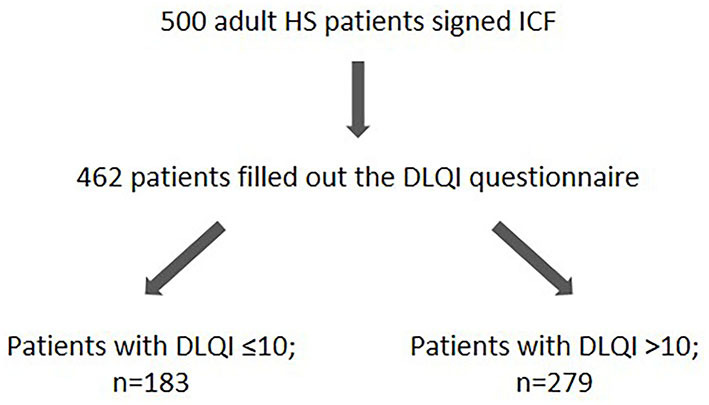
Overview on the HS study population. The number of HS study participants as well as the number of patients showing a DLQI score ≤ 10 and DLQI > 10 are given. DLQI, Dermatology Life Quality Index; HS, hidradenitis suppurativa; ICF, informed consent form.

A non-interventional, cross-sectional, mono-centric study with 500 patients suffering from HS was conducted. The patients: (i) visited the Department of Dermatology, Venereology, and Allergology, University Hospital Charité, Berlin, Germany, from February 2012 to November 2017, (ii) provided written informed consent, and (iii) fulfilled the following inclusion criteria: at least 18 years of age and diagnosis of HS. The diagnosis of HS was made by an experienced dermatologist on the basis of clinical presentation according to the diagnostic criteria (26). QoL data from 462 patients (278 women, 184 men) were available, evaluated, and are presented in this manuscript (Figure 1). Missing data were not replaced for analysis. The number of patients that gave information about specific parameters is indicated in the figure legends.

The study was conducted according to the principles expressed in the Declaration of Helsinki. The written informed consent was obtained from all participants and the study was approved by the clinical institutional review board of Charité University Hospital, Berlin.

### Patients' Characteristics

The QoL was analyzed using the Dermatology Life Quality Index (DLQI; see below). Demographic characteristics, family history, details of the course of HS (e.g., age at onset), clinical data (e.g., blood pressure), details of affected regions, and blood cell counts were also collected. The body mass index (BMI) of each patient was calculated as the weight (kg)/height (m)^2^. Disease severity was assessed using Hurley's 3-stage scale and the Sartorius score. Higher scores indicate greater severity of disease. The most important demographic and clinical characteristics are included in Table 1.

**Table 1:** Demographic and clinical characteristics of the study cohort (*n* = 462).

	**HS patients**
**Age in years**	
(Mean ± SD)	38.8 ± 10.9
(Range)	18.0–78.4
**Sex**	
Females (%)	60.2
Males (%)	39.8
**BMI**	
(Mean ± SD)	28.9 ± 5.9
(Range)	17.2–52.6
**Smoking habits**	
Smoker (%)	66.5
Ex-smoker (%)	18.7
Never smoker (%)	14.8
**Disease duration, years**	
(Mean ± SD)	13.4 ± 9.8
(Range)	0.1–52.9
**Sartorius score**	
(Mean ± SD)	49.2 ± 34.7
(Range)	0–216
**Hurley score**	
(Mean ± SD)	1.64 ± 0.89
(Range)	0–3
**Family history of HS**	
Positive (%)	33.8
Negative (%)	66.2

*BMI, body mass index; HS, hidradenitis suppurativa; SD, standard deviation*.

### Dermatology Life Quality Index

DLQI is a self-administered questionnaire that was developed to assess QoL of patients suffering from skin diseases. The questionnaire consists of 10 questions covering six various aspects of life: symptoms and feelings, daily activities, leisure, job and school, personal relationships, and treatment of the disease. Each question is scored from 0 to 3, and the sum of the individual values provides the total score used for evaluation: 0 to 1 = no effect on patient's life, 2–5 = small effect, 6–10 = moderate effect, 11–20 = very large effect, and 21–30 = extremely large effect.

### Statistical Analysis

Statistical calculations were performed using SPSS software (IBM, Ehningen, Germany). Continuous variables were described as means ± standard deviation (SD) or standard error of the mean (SEM). The Mann-Whitey-*U*-test (two-tailed) was used to compare means. Discontinuous variables were described using percentages of each modality and were analyzed using the Chi-square test. Correlation analyses were performed by means of Spearman's rank correlation test. Statistical significance was achieved if *P* < 0.05 (^*^P < 0.05; ^**^P ≤ 0.01; ^***^P ≤ 0.001).

## Results

### QoL Impairment

HS is a very complex disease that has a relevant genetic component and involves a physical as well as a psychological level of suffering with a significant mutual influence. To gain a deeper insight into these interactions, we first investigated the impact of HS on the QoL in a German patient cohort comprised of 500 HS patients (Figure 1).

**Figure 2:**
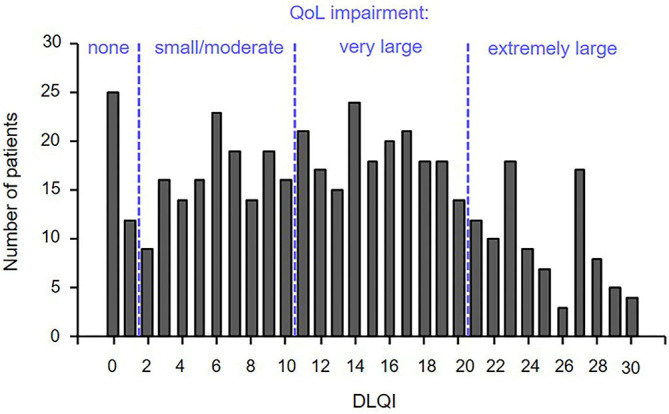
More than half of the patients with HS showed a severe QoL impairment. QoL was assessed by means of the DLQI questionnaire in 462 patients with HS. The numbers of patients scoring a certain DLQI value is indicated. DLQI category: 0–1 = no effect on patient's life, 2–10 = small / moderate effect, 11–20 = very large effect, and 21–30 = extremely large effect. DLQI, Dermatology Life Quality Index; QoL, quality of life.

The detected mean (± SEM) DLQI score of all evaluated HS patients (*n* = 462) was 13.18 (± 0.37), indicating that HS has a profound effect on the lives of patients. A very large (DLQI >10 and ≤20) impairment in QoL was noted in 40.3% of patients, whereas 20.1% of patients had an extremely large (DLQI > 20) impairment (Figure 2). Highest disturbances in patients' QoL were noted in the context of symptoms and feelings (Table 2). In fact, questions focusing on the presence of symptoms like pain, soreness, stinging, or itching (1.56 ± 0.04) and embarrassment or restrictions in self-consciousness (1.57 ± 0.05) achieved the highest score value, indicating the largest impairment.

**Table 2:** Mean scores for each DLQI question.

	**DLQI question**	**Mean ± SEM**
1	How itchy, sore, painful or stinging has your skin condition been?	1.56 ± 0.04
2	How embarrassed or self-conscious have you been because of your skin?	1.57 ± 0.05
3	How much has your skin interfered with you going shopping or looking after your home or garden?	1.28 ± 0.05
4	How much has your skin influenced the clothes you wear?	1.53 ± 0.05
5	How much has your skin affected your social or leisure activities?	1.21 ± 0.05
6	How much has your skin made it difficult for you to do any sport?	1.38 ± 0.06
7	Has your skin prevented you from working or studying?	1.22 ± 0.06
8	How much has your skin created problems with your partner or any of your close friends or relatives?	0.90 ± 0.05
9	How much has your skin caused any sexual difficulties?	1.29 ± 0.06
10	How much of a problem has the treatment for your skin been?	1.26 ± 0.05

*DLQI, Dermatology Life Quality Index; SEM, standard error of the mean*.

### QoL Impairment Correlates With Severity of HS

**Figure 3:**
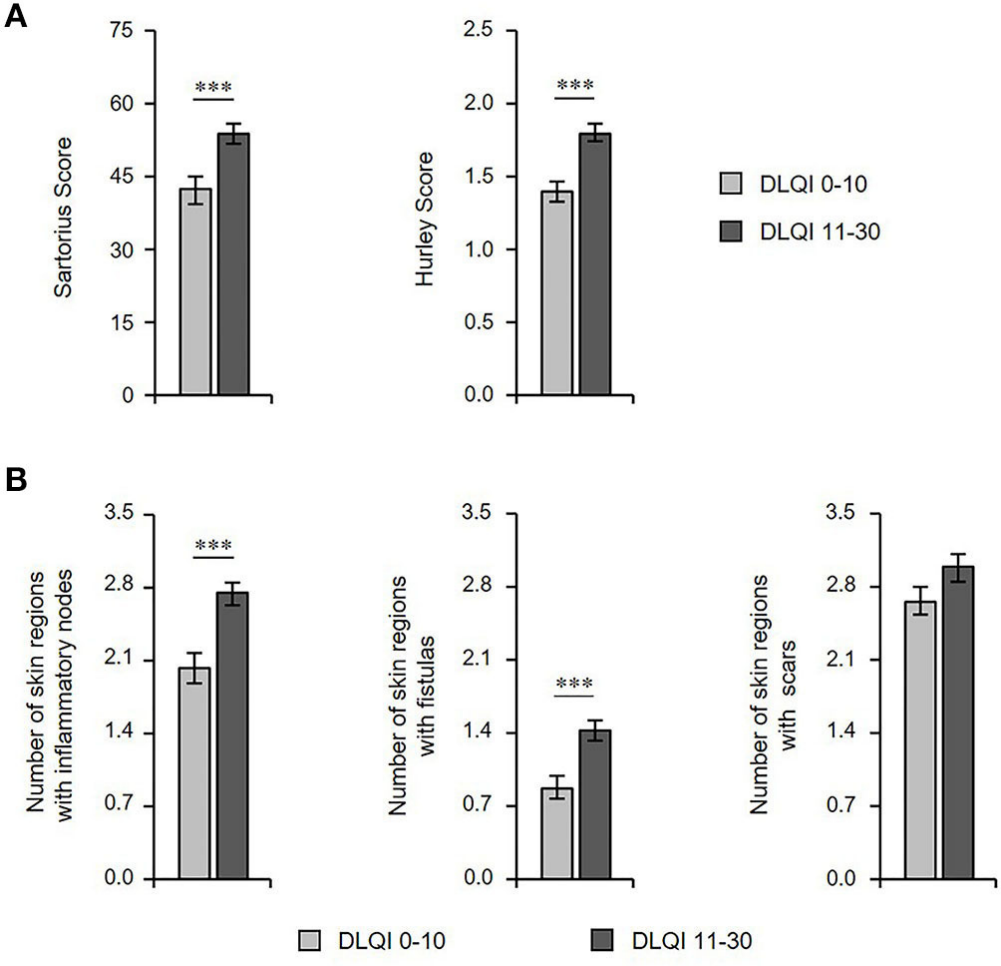
Extent of HS cutaneous alterations correlates with QoL impairment. **(A)** The disease severity (Sartorius score, *n* = 418; Hurley score, *n* = 411), and **(B)** number of skin regions with inflammatory nodes, fistulas, and scars (*n* = 415) in patients with HS with small/moderate (DLQI ≤ 10) and very/extremely large (DLQI > 10) QoL impairment are indicated as mean ± SEM. The significance of differences was assessed using the Mann-Whitney U test (two-tailed; ***P ≤ 0.001). DLQI, Dermatology Life Quality Index.

Next, we aimed to determine whether there is an association between the clinical manifestation of the disease and QoL. As assumed, the extent of impairment of QoL positively correlated with disease severity, as assessed by the Sartorius score (*r*_s_ = 0.307; *P* = 0.000) and the Hurley score (*r*_s_ = 0.273; *P* = 0.000; Table 3). Accordingly, we found a significant difference in disease severity between patients with small/moderate (DLQI ≤ 10) vs. very/extremely large (DLQI > 10) QoL impairment (Figure 3A). In more detailed analyses, we included information about the potential involvement of right and left axillary, inguinal, and gluteal areas as well as pilonidal sinus. These analyses revealed that DLQI was significantly associated with the number of affected body regions (*r*_s_ = 0.253; *P* = 0.000; Table 3). Furthermore, there was also a significant difference in the number of these regions containing nodules or fistulas (but not scars) between patients with small/moderate vs. large QoL impairment (Figure 3B).

**Table 3:** Correlations between DLQI and selected clinical data.

	**Correlation with DLQI**
Sartorius score	0.307 (**0.000**)
Hurley score	0.273 (**0.000**)
Number of involved regions	0.253 (**0.000**)
Blood leukocyte count	0.274 (**0.000**)
Age at onset (years)	−0.007 (0.888)
Age (years)	0.044 (0.345)
BMI	0.167 (**0.000**)
Waist circumference	0.158 (**0.002**)

*BMI, body mass index; DLQI, Dermatology Life Quality Index*.

*The data were tested for correlation using the Spearman's rank correlation analysis. For each field, the Spearman's rank correlation coefficients (and P-values) are indicated. Significant P-values (P < 0.050) are in boldface*.

### Localization of Lesions Influences QoL Impairment

**Figure 4:**
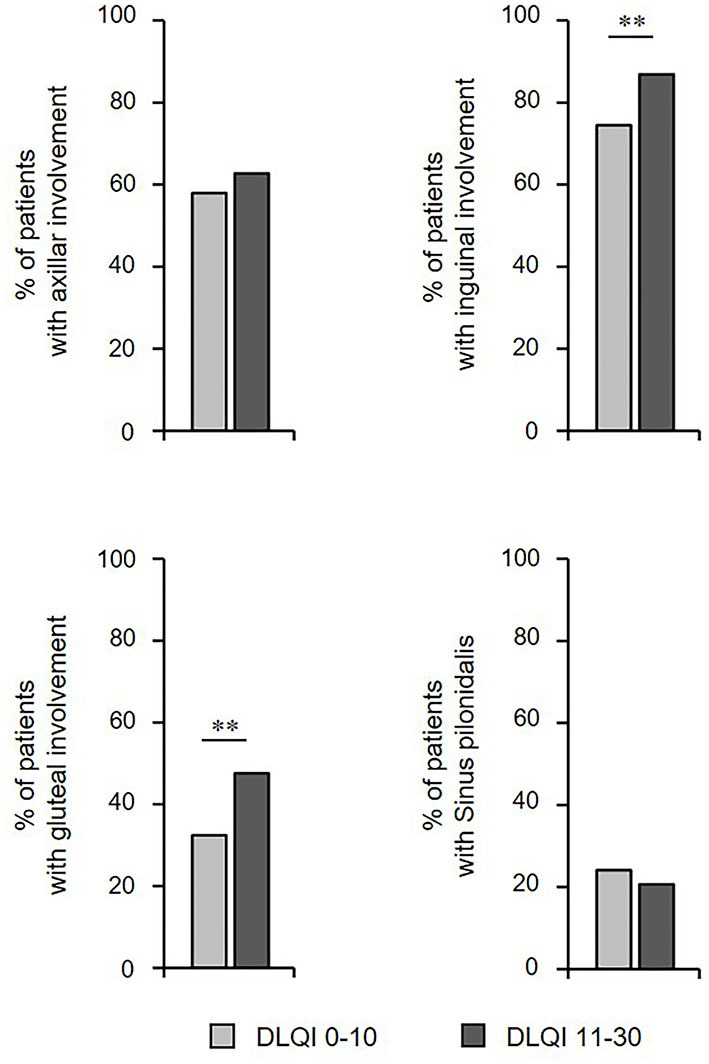
Localization of skin lesions influences QoL. The frequency of HS patients (*n* = 415) showing involvement of a specific localization of skin alterations and small/moderate (DLQI ≤ 10) vs. very/extremely large (DLQI > 10) QoL impairment are provided. The significance of differences was assessed using the Chi-square test (**P ≤ 0.01). DLQI, Dermatology Life Quality Index.

We further examined whether there were any differences in the QoL impairment in relation to body sites of clinical manifestation. Interestingly, patients with more profound reduction in QoL had significantly more frequent HS skin alterations at inguinal and gluteal sites (Figure 4). Conversely, no significant differences for axillary site or pilonidal sinus involvement were determined between patients with small/moderate (DLQI ≤ 10) vs. very/extremely large (DLQI > 10) QoL impairment (Figure 4).

### Impairment of QoL and HS Comorbidities

**Figure 5:**
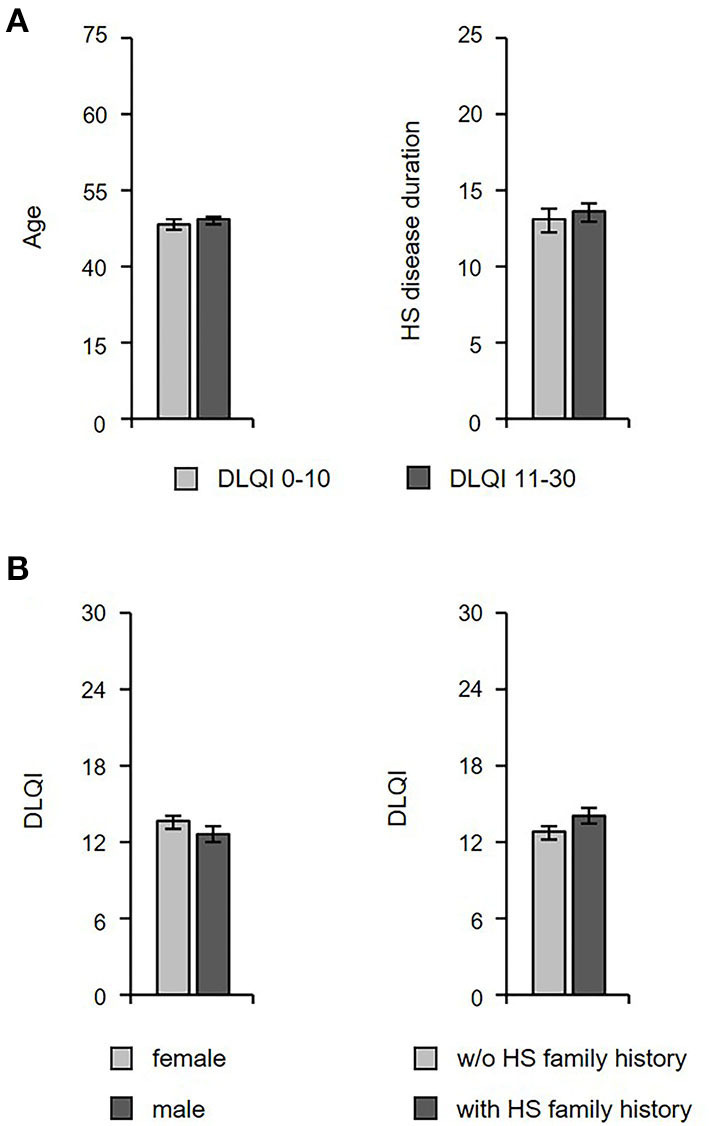
The QoL impairment is not related to the age, disease duration, and family history of HS patients. **(A)** The age (*n* = 462) and HS disease duration (*n* = 440) in HS patients with small/moderate (DLQI ≤ 10) vs. very/extremely large (DLQI > 10) QoL impairment is given as mean ± SEM. **(B)** DLQI scores among female and male HS patients (left panel; *n* = 462) as well as patients with or without a HS family history (right panel; *n* = 459) are given as mean ± SEM. DLQI, Dermatology Life Quality Index.

In the second part of our study, we investigated whether there are further characteristics of HS patients that are associated with a QoL impairment. The patients' age and HS disease duration seem to have no actual impact on QoL, as no difference in these parameters between patients with small/moderate (DLQI ≤ 10) vs. very/extremely large (DLQI > 10) QoL impairment was detected (Figure 5A). Moreover, there were no differences in DLQI scores between patients with vs. those without positive family history for HS or between female and male HS patients (Figure 5B).

**Figure 6:**
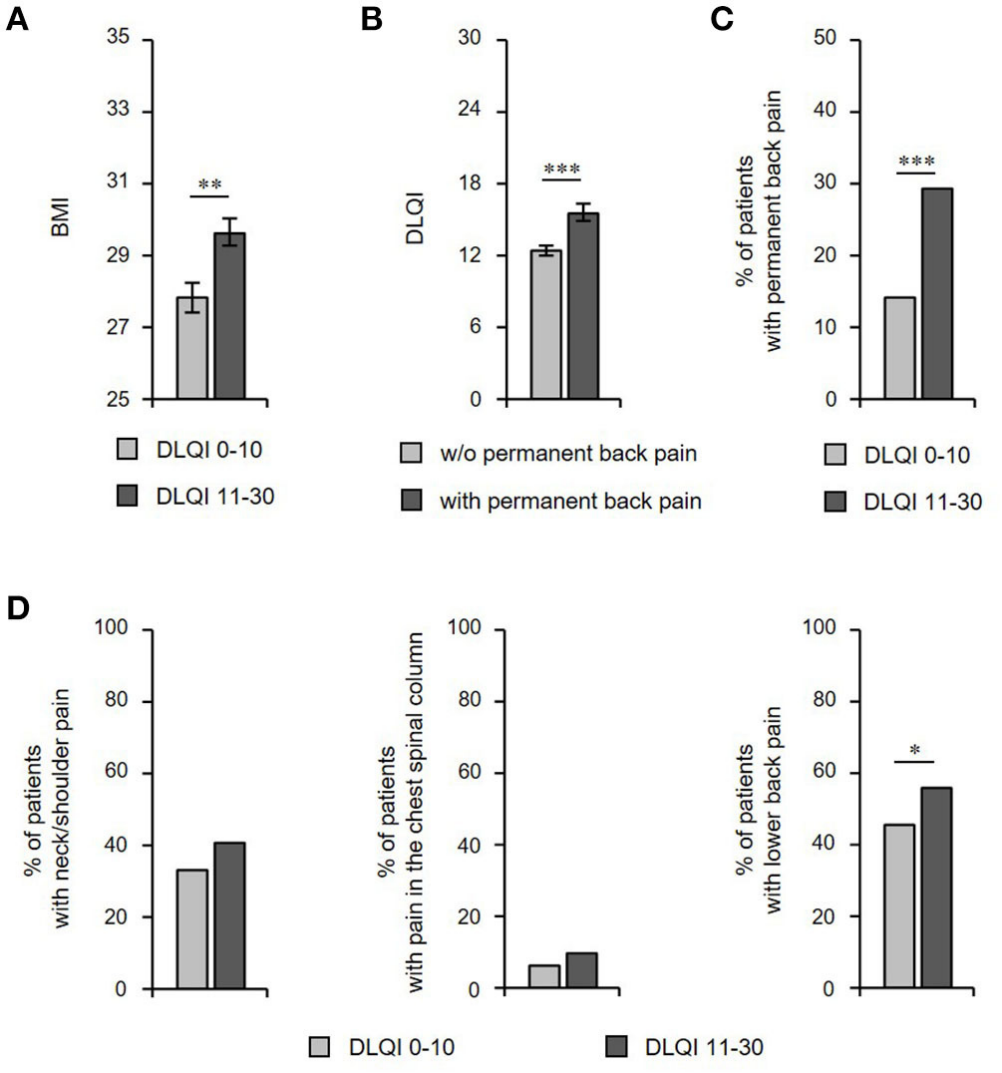
Impairment of QoL is associated with HS comorbidities. **(A)** BMI (*n* = 437) in HS patients with small/moderate (DLQI ≤ 10) vs. very/extremely large (DLQI > 10) QoL impairment is given as mean ± SEM. The significance of differences was assessed using the Mann-Whitney U test (two-tailed; **P ≤ 0.01). **(B)** DLQI scores of HS patients with or without very frequent/permanent back pain are presented as mean ± SEM (*n* = 462). The significance of differences was assessed using the Mann-Whitney U test (***P < 0.001). **(C)** The frequency of HS patients (*n* = 462) with very frequent/permanent back pain in patients with small/moderate (DLQI ≤ 10) vs. very/extremely large (DLQI > 10) QoL impairment is provided. The significance of differences was assessed using the Chi-square test (***P ≤ 0.001). **(D)** The frequency of HS patients (*n* = 462) showing pain of neck/shoulder (left), chest spinal column (middle), and lower back (right) among patients with small/moderate (DLQI ≤ 10) vs. very/extremely large (DLQI > 10) QoL impairment is provided. The significance of differences was assessed using the Chi-square test (*P < 0.05). BMI, body mass index; DLQI, Dermatology Life Quality Index.

However, our analyses revealed a significant association between QoL and BMI (*r*_s_ = 0.167; *P* = 0.000), as well as between QoL and waist circumference (*r*_s_ = 0.158; *P* = 0.002; Table 3). We found a larger waist circumference (for women 95.2 ± 2.0 vs. 101.2 ± 1.4; *P* = 0.023 / for men 100.3 ± 1.6 vs. 105.8 ± 2.2; *P* = 0.066) and a higher BMI (Figure 6A) in patients with very/extremely large vs. small/moderate QoL impairment. Accordingly, a significant difference in DLQI score between patients with BMI <25 and BMI ≥ 30 (11.61 ± 0.71 vs. 14.98 ± 0.62; *P* = 0.000) was detected. Furthermore, we discovered that HS patients with very frequent/permanent back pain had larger QoL impairment (Figure 6B). On the other side, the frequency of patients with very frequent/permanent back pain was significantly higher among patients with very/extremely large compared to those with small/moderate QoL impairment (Figure 6C). Interestingly, when considering the location of back pain, the patients with very/extremely large QoL impairment suffered more frequently from lower back pain (Figure 6D).

### DLQI and Treatment

**Figure 7:**
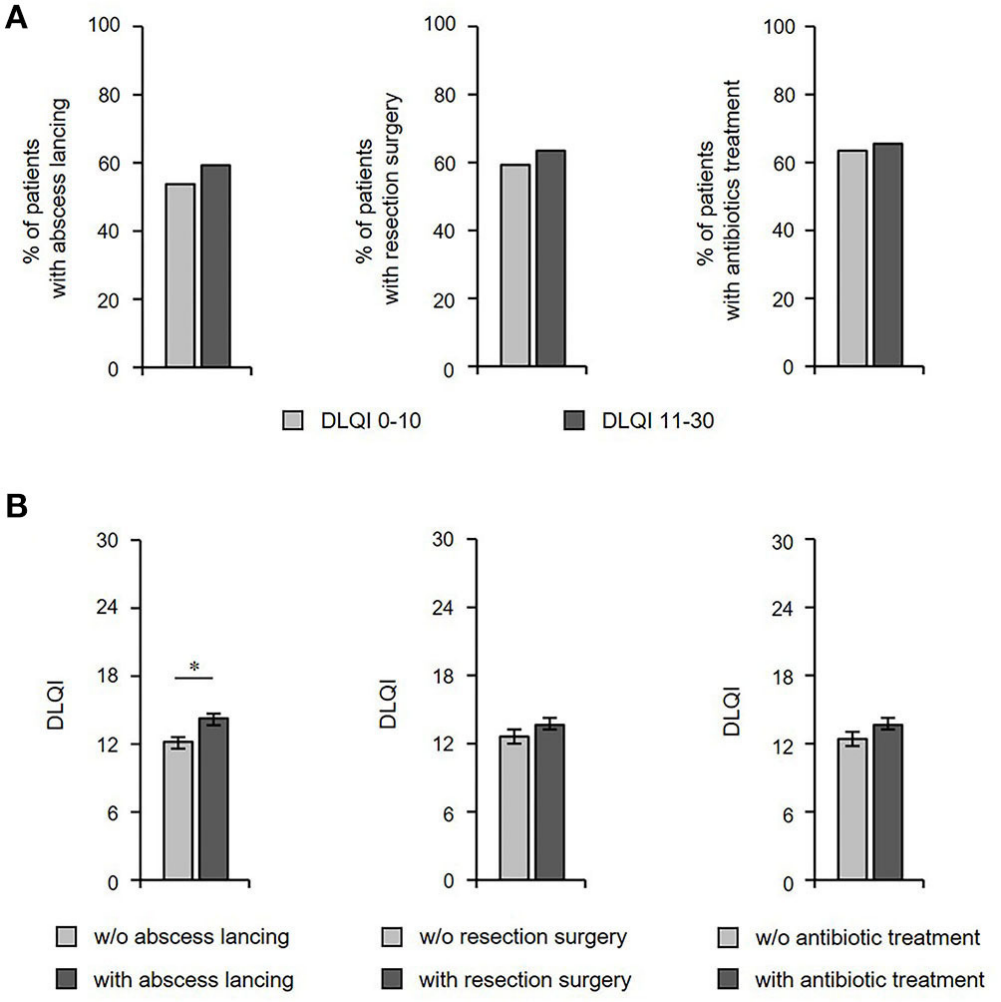
DLQI scores are not associated with current type of treatment. **(A)** The frequency of HS patients (*n* = 435) who had received therapeutic abscess lancing (left), resection surgery (middle) and antibiotic treatment (right) in patients with small/moderate (DLQI ≤ 10) vs. very/extremely large (DLQI > 10) QoL impairment is provided. **(B)** DLQI scores of patients with HS who have or have not undergone the respective therapeutic regimen are indicated using mean ± SEM (*n* = 435). The significance of differences was assessed using the Mann-Whitney U test (*P < 0.05). DLQI, Dermatology Life Quality Index.

We were also interested in determining whether the prescribed therapeutic regimen had any implications on QoL. Surprisingly, there were no significant differences in terms of the proportion of patients that had previously undergone abscess lancing, resection surgery, or antibiotic treatment between patients with small/moderate vs. very/extremely large QoL impairment (Figure 7A). Accordingly, there were no significant differences in DLQI scores between patients who had vs. those who had not undergone resection surgery and between patients who have been prescribed antibiotic treatment vs. those who were left without those treatments (Figure 7B). Interestingly, our analyses even showed that the QoL impairment was even larger in the group undergone abscess lancing in the past (Figure 7B).

## Discussion

Hidradenitis suppurativa is a common chronic disease affecting the intertriginous skin areas. Previously, the Jemec group and the Szepietowski group indicated that HS has a negative impact on QoL (20, 22). Our results, based on a larger number of patients, suggest that now, 10–20 years later, HS still leads to a profound impairment in the QoL of individuals afflicted with the disease. In fact, HS has a large or extremely large negative effect on QoL in ~60% of patients. Importantly, patients with HS have a significantly lower QoL compared to patients with other chronic skin diseases (Table 4), including alopecia areata (27, 28), acne vulgaris (29, 30), vitiligo (31, 32), atopic eczema (33, 34), psoriasis vulgaris (35, 36), and non-melanoma skin cancer (37, 38).

**Table 4:** DLQI scores reported for different skin disorders.

**Disease**	**HS**	**Alopecia areata**	**Acne vulgaris**	**Vitiligo**	**Atopic eczema**	**Psoriasis vulgaris**	**NMSC**
DLQI score (mean ± SD)	13.18 ± 7.99	6.3 ± 6.3	5.1 ± 4.2	4.3 ± 4.9	6.6 ± 5.4	6.9 ± 6.3	2.4 ± 2.7
		7.9 ± 7.6	8.2 ± 4.8	5.2 ± 5.4	8.3 ± 5.9	10.8 ± 7.1	4.1 ± 4.3
References	Current study	(27, 28)	(29, 30)	(31, 32)	(33, 34)	(35, 36)	(37, 38)

*DLQI, Dermatology Life Quality Index; HS, hidradenitis suppurativa; NMSC, non-melanoma skin cancer; SD, standard deviation*.

Jemec *et al*. noted that the highest proportion of physical disability resulted from the soreness and pain induced by HS (20). In fact, the presence of pain but not odor seems to be a crucial contributor to QoL impairment (39, 40), and pain severity correlated significantly with DLQI (41, 42). Moreover, embarrassment and diminished self-consciousness led to a considerable extent of QoL impairment in our patient cohort. Furthermore, the restriction in the choice of clothing due to skin inflammation appears to be another relevant problem. That, in turn might have a negative impact on self-realization and thereby self-consciousness.

Jemec *et al*. and Matusiak *et al*. also determined that QoL impairment clearly correlates with the severity of skin alterations including the number of lesions (20, 22), a fact that was confirmed by further studies (43–45) and was also observed in our study involving a large patient cohort. In this context, a very recently published study showed a positive correlation of QoL impairment with IHS4 (42). Moreover, we also found that anogenital localization of HS skin alterations has a substantial negative impact on QoL, an observation that was very recently published by Jørgensen *et al*. (45). This localization may have a profound effect not only on the psychological comfort of patients with HS, but also on common aspects of physical impairment (e.g., profound pain, malodorous secretion, and limited mobility). Our detailed analyses of the association between QoL impairment and disease severity revealed that nodules or fistulas, but not scars, have a large negative impact on QoL.

Our study indicates that there is an association between QoL impairment and HS comorbidities. In fact, we did not only detect a positive correlation between DLQI score and BMI as well as waist circumference, but we also found a higher BMI in patients with a large QoL impairment. Furthermore, we detected a significant difference with regards to back pain between patients with small/moderate vs. large QoL reduction. These results were related to lower back pain, in particular. It should be noted that there was no association between QoL impairment and patients' age or a positive family history for HS.

Interestingly, there were no differences between patients with small/moderate vs. large QoL impairment with regards to classical therapeutic procedures (resection surgery, antibiotic treatment) in our study. These data suggest that classical treatment options for HS do not lead to a long-lasting improvement in the patient's QoL. This highlights the necessity of a consequent implementation of the recently approved HS therapy, which is anti-TNF-α antibody. In fact, adalimumab and its biosimilars are approved for the treatment of moderate to severe HS and adalimumab has been shown to improve QoL in clinical studies as well as in clinical practice (46). Moreover, further research in HS is needed to develop new treatments that provide more effective relief for patients with this distressing, extremely debilitating disease. In this context, we firmly believe that offering psychological guidance to these patients may help improve acceptance of the skin condition and ameliorate therapeutic results.

In summary, our study revealed that HS causes profound impairment in patients' QoL. The degree of this impairment correlated with the severity of skin alterations, in particular, with the number of affected regions, anogenital localization, and the presence of nodules and fistulas. Furthermore, QoL reduction was associated with elevated BMI and back pain. In view of the extent of QoL impairment in patients with HS, we recommend implementing the DLQI instrument when deciding on the appropriate treatment strategy and in assessing the therapeutic response.

## Data Availability Statement

The main data are presented within tables and figures of the article. Further data will be made available upon request according to the legal possibilities by the corresponding author.

## Ethics Statement

The study was conducted according to the principles expressed in the Declaration of Helsinki. The study was approved by the clinical institutional review board (Ethikkommission) of Charité University Hospital, Berlin, Germany. Written informed consent was obtained from all participants.

## Author Contributions

SS-B: design of the study, conceptual idea of the manuscript, data collection and analysis, and drafting of the manuscript. AT: data collection and analysis and drafting of the manuscript. SB, JH-M, and BF: conceptual idea of the manuscript and revision of the manuscript. KWi: contribution to data curation, revision of the manuscript, and editing of manuscript. KWo: visualization of the results and revision of the manuscript. RS: conceptual idea of the manuscript, statistical analysis, and revision of the manuscript. All authors contributed to the article and approved the submitted version.

## Conflict of Interest

SS-B has received research grants, or honoraria for participation in advisory boards, clinical trials or as speaker for one or more of the following: AbbVie, Biogen IDEC, La Roche-Posay, Novartis Pharma, Parexel, and UCB Pharma. SB and JH-M were employees of AbbVie Deutschland GmbH & Co. KG at the time of publication development, may own AbbVie stock, and are currently employed at Sanofi Aventis Deutschland GmbH. KWo has received research grants, travel grants, consulting honoraria or lecturer's honoraria from AbbVie, Biogen IDEC, Celgene, Charité Research Organisation, Dr. Willmar Schwabe, Flexopharm, Janssen- Cilag, Novartis Pharma, Pfizer, Sanofi-Aventis, TFS Trial Form Support, and UCB Pharma. BF is an employee of AbbVie and may own AbbVie stock. RS has received research grants, scientific awards, or honoraria for participation in advisory boards, clinical trials or as speaker for one or more of the following: AbbVie, AMGEN, Bayer, Biogen IDEC, Boehringer Ingelheim Pharma, Celgene, Charité Research Organisation, CSL Behring, Dr. Willmar Schwabe, Flexopharm, Janssen-Cilag, La Roche-Posay Laboratoire Dermatologique, Novartis Pharma, Parexel, Pfizer, Sanofi-Aventis, TFS Trial Form Support, and UCB Pharma. The evaluation of patient data was partly supported by AbbVie Deutschland GmbH & Co. KG. AbbVie Deutschland GmbH & Co. KG contributed in writing, reviewing, and approval of the final version. No honoraria or payments were made for authorship. The remaining authors declare that the research was conducted in the absence of any commercial or financial relationships that could be construed as a potential conflict of interest.

## Abbreviations

BMI: body mass index
DLQI: Dermatology Life Quality Index
HS: hidradenitis suppurativa
QoL: quality of life.

## References

[1] Sabat R Jemec GBE Matusiak L Kimball AB Prens E Wolk K. Hidradenitis suppurativa. Nat Rev Dis Primers. (2020) 6:18. 10.1038/s41572-020-0149-1 32165620

[2] Jemec GB Heidenheim M Nielsen NH. The prevalence of hidradenitis suppurativa and its potential precursor lesions. J Am Acad Dermatol. (1996) 35:191–4. 10.1016/S0190-9622(96)90321-7 8708018

[3] Ingram JR. The epidemiology of hidradenitis suppurativa. Br J Dermatol. (2020) 183:990–8. 10.1111/bjd.19435 32880911

[4] Sachdeva M Shah M Alavi A. Race-specific prevalence of hidradenitis suppurativa. J Cutan Med Surg. (2021) 25:177–87. 10.1177/1203475420972348 33174482

[5] Schneider-Burrus S Lux G van der Linde K Barbus S Huss-Marp J Tsaousi A. Hidradenitis suppurativa - prevalence analyses of German statutory health insurance data. J Eur Acad Dermatol Venereol. (2021) 35:e32–5. 10.1111/jdv.16783 32580237

[6] Wolk K Join-Lambert O Sabat R. Aetiology and pathogenesis of hidradenitis suppurativa. Br J Dermatol. (2020) 183:999–1010. 10.1111/bjd.19556 33048349

[7] Del Duca E Morelli P Bennardo L Di Raimondo C Nistico SP. Cytokine pathways and investigational target therapies in hidradenitis suppurativa. Int J Mol Sci. (2020) 21:8436. 10.3390/ijms21228436 33182701 PMC7696820

[8] von Laffert M Helmbold P Wohlrab J Fiedler E Stadie V Marsch WC. Hidradenitis suppurativa (acne inversa): early inflammatory events at terminal follicles and at interfollicular epidermis. Exp Dermatol. (2010) 19:533–7. 10.1111/j.1600-0625.2009.00915.x 19659829

[9] Wolk K Warszawska K Hoeflich C Witte E Schneider-Burrus S Witte K. Deficiency of IL-22 contributes to a chronic inflammatory disease: pathogenetic mechanisms in acne inversa. J Immunol. (2011) 186:1228–39. 10.4049/jimmunol.0903907 21148041

[10] Hessam S Sand M Gambichler T Skrygan M Ruddel I Bechara FG. Interleukin-36 in hidradenitis suppurativa: evidence for a distinctive proinflammatory role and a key factor in the development of an inflammatory loop. Br J Dermatol. (2018) 178:761–7. 10.1111/bjd.16019 28975626

[11] Scala E Di Caprio R Cacciapuoti S Caiazzo G Fusco A Tortorella E. A new T helper 17 cytokine in hidradenitis suppurativa: antimicrobial and proinflammatory role of interleukin-26. Br J Dermatol. (2019) 181:1038–45. 10.1111/bjd.17854 30829398

[12] Wolk K Brembach TC Simaite D Bartnik E Cucinotta S Pokrywka A. Activity and components of the G-CSF pathway in hidradenitis suppurativa. Br J Dermatol. (2021). 10.1111/bjd.19795. [Epub ahead of print]. 33400270

[13] Witte-Handel E Wolk K Tsaousi A Irmer ML Mossner R Shomroni O. The IL-1 pathway is hyperactive in hidradenitis suppurativa and contributes to skin infiltration and destruction. J Invest Dermatol. (2019) 139:1294–305. 10.1016/j.jid.2018.11.018 30528824

[14] Matusiak L Szczech J Bieniek A Nowicka-Suszko D Szepietowski JC. Increased interleukin (IL)-17 serum levels in patients with hidradenitis suppurativa: implications for treatment with anti-IL-17 agents. J Am Acad Dermatol. (2017) 76:670–5. 10.1016/j.jaad.2016.10.042 28041632

[15] Wolk K Wenzel J Tsaousi A Witte-Handel E Babel N Zelenak C. Lipocalin-2 is expressed by activated granulocytes and keratinocytes in affected skin and reflects disease activity in acne inversa/hidradenitis suppurativa. Br J Dermatol. (2017) 177:1385–93. 10.1111/bjd.15424 28256718

[16] Schneider-Burrus S Witte-Haendel E Christou D Rigoni B Sabat R Diederichs G. High prevalence of back pain and axial spondyloarthropathy in patients with hidradenitis suppurativa. Dermatology. (2016) 232:606–12. 10.1159/000448838 27649417

[17] Rondags A van Straalen KR Arends S van der Zee HH Prens EP Spoorenberg A. High prevalence of clinical spondyloarthritis features in patients with hidradenitis suppurativa. J Am Acad Dermatol. (2019) 80:551–4 e551. 10.1016/j.jaad.2018.06.028 30554892

[18] Sabat R Chanwangpong A Schneider-Burrus S Metternich D Kokolakis G Kurek A. Increased prevalence of metabolic syndrome in patients with acne inversa. PLoS ONE. (2012) 7:e31810. 10.1371/journal.pone.0031810 22359634 PMC3281019

[19] Tiri H Jokelainen J Timonen M Tasanen K Huilaja L. Substantially reduced life expectancy in patients with hidradenitis suppurativa: a finnish nationwide registry study. Br J Dermatol. (2019) 180:1543–4. 10.1111/bjd.17578 30597518

[20] von der Werth JM Jemec GB. Morbidity in patients with hidradenitis suppurativa. Br J Dermatol. (2001) 144:809–13. 10.1046/j.1365-2133.2001.04137.x 11298541

[21] Wolkenstein P Loundou A Barrau K Auquier P Revuz J Quality of Life Group of the French Society of D. Quality of life impairment in hidradenitis suppurativa: a study of 61 cases. J Am Acad Dermatol. (2007) 56:621–3. 10.1016/j.jaad.2006.08.061 17097366

[22] Matusiak L Bieniek A Szepietowski JC. Psychophysical aspects of hidradenitis suppurativa. Acta Derm Venereol. (2010) 90:264–8. 10.2340/00015555-0866 20526543

[23] Kurek A Peters EM Chanwangpong A Sabat R Sterry W Schneider-Burrus S. Profound disturbances of sexual health in patients with acne inversa. J Am Acad Dermatol. (2012) 67:422–8, 428 e421. 10.1016/j.jaad.2011.10.024 22182915

[24] Kurek A Johanne Peters EM Sabat R Sterry W Schneider-Burrus S. Depression is a frequent co-morbidity in patients with acne inversa. J Dtsch Dermatol Ges. (2013) 11:743–9, 743–50. 10.1111/ddg.12067 23565584

[25] Matusiak L. Profound consequences of hidradenitis suppurativa: a review. Br J Dermatol. (2020) 183:e171–7. 10.1111/bjd.16603 29744872

[26] Zouboulis CC Tzellos T Kyrgidis A Jemec GBE Bechara FG Giamarellos-Bourboulis EJ. Development and validation of the International Hidradenitis Suppurativa Severity Score System (IHS4), a novel dynamic scoring system to assess HS severity. Br J Dermatol. (2017) 177:1401–9. 10.1111/bjd.15748 28636793

[27] Zhang M Zhang N. Quality of life assessment in patients with alopecia areata and androgenetic alopecia in the People's Republic of China. Patient Prefer Adherence. (2017) 11:151–5. 10.2147/PPA.S121218 28203058 PMC5293494

[28] Abedini R Hallaji Z Lajevardi V Nasimi M Karimi Khaledi M Tohidinik HR. Quality of life in mild and severe alopecia areata patients. Int J Womens Dermatol. (2018) 4:91–4. 10.1016/j.ijwd.2017.07.001 29872683 PMC5986230

[29] Richter C Trojahn C Hillmann K Dobos G Kanti V Vogt A. Sensitivity to change of the Dermatology Life Quality Index in adult females with facial acne vulgaris: a validation study. J Eur Acad Dermatol Venereol. (2017) 31:169–74. 10.1111/jdv.13757 27393576

[30] Ghaderi R Saadatjoo A Ghaderi F. Evaluating of life quality in patients with acne vulgaris using generic and specific questionnaires. Dermatol Res Pract. (2013) 2013:108624. 10.1155/2013/108624 24371434 PMC3859265

[31] Ingordo V Cazzaniga S Medri M Raone B Digiuseppe MD Musumeci ML. To what extent is quality of life impaired in vitiligo? A multicenter study on Italian patients using the dermatology life quality index. Dermatology. (2014) 229:240–7. 10.1159/000363407 25358871

[32] Morales-Sanchez MA Vargas-Salinas M Peralta-Pedrero ML Olguin-Garcia MG Jurado-Santa Cruz F. Impact of vitiligo on quality of life. Actas Dermosifiliogr. (2017) 108:637–42. 10.1016/j.adengl.2017.06.001 28456327

[33] Kiebert G Sorensen SV Revicki D Fagan SC Doyle JJ Cohen J. Atopic dermatitis is associated with a decrement in health-related quality of life. Int J Dermatol. (2002) 41:151–8. 10.1046/j.1365-4362.2002.01436.x 12010340

[34] Dieris-Hirche J Gieler U Petrak F Milch W Te Wildt B Dieris B. Suicidal ideation in adult patients with atopic dermatitis: a German cross-sectional study. Acta Derm Venereol. (2017) 97:1189–95. 10.2340/00015555-2741 28676884

[35] Augustin M Langenbruch A Gutknecht M Reich K Korber A Maassen D. Definition of psoriasis severity in routine clinical care: current guidelines fail to capture the complexity of long-term psoriasis management. Br J Dermatol. (2018) 179:1385–91. 10.1111/bjd.17128 30334253

[36] Schmitt J Kuster D. Correlation between Dermatology Life Quality Index (DLQI) scores and Work Limitations Questionnaire (WLQ) allows the calculation of percent work productivity loss in patients with psoriasis. Arch Dermatol Res. (2015) 307:451–3. 10.1007/s00403-015-1567-x 25940274

[37] Rhee JS Matthews BA Neuburg M Smith TL Burzynski M Nattinger AB. Skin cancer and quality of life: assessment with the Dermatology Life Quality Index. Dermatol Surg. (2004) 30:525–9. 10.1097/00042728-200404000-00013 15056143

[38] Abedini R Nasimi M Noormohammad Pour P Moghtadaie A Tohidinik HR. Quality of life in patients with non-melanoma skin cancer: implications for healthcare education services and supports. J Cancer Educ. (2019) 34:755–9. 10.1007/s13187-018-1368-y 29705894

[39] Matusiak L Szczech J Kaaz K Lelonek E Szepietowski JC. Clinical characteristics of pruritus and pain in patients with hidradenitis suppurativa. Acta Derm Venereol. (2018) 98:191–4. 10.2340/00015555-2815 28971209

[40] Alavi A Farzanfar D Lee RK Almutairi D. The contribution of malodour in quality of life of patients with hidradenitis suppurativa. J Cutan Med Surg. (2018) 22:166–74. 10.1177/1203475417745826 29231053

[41] Frings VG Bauer B Gloditzsch M Goebeler M Presser D. Assessing the psychological burden of patients with hidradenitis suppurativa. Eur J Dermatol. (2019) 29:294–301. 10.1684/ejd.2019.3552 31145081

[42] Krajewski PK Matusiak L von Stebut E Schultheis M Kirschner U Nikolakis G. Quality-of-life impairment among patients with hidradenitis suppurativa: a cross-sectional study of 1795 patients. Life. (2021) 11:34. 10.3390/life11010034 33429896 PMC7828046

[43] Alavi A Anooshirvani N Kim WB Coutts P Sibbald RG. Quality-of-life impairment in patients with hidradenitis suppurativa: a Canadian study. Am J Clin Dermatol. (2015) 16:61–5. 10.1007/s40257-014-0105-5 25432664

[44] Kluger N Ranta M Serlachius M. The burden of hidradenitis suppurativa in a cohort of patients in Southern Finland: a pilot study. Skin Appendage Disord. (2017) 3:20–7. 10.1159/000455236 28611997 PMC5465670

[45] Jorgensen AR Holm JG Ghazanfar MN Yao Y Ring HC Thomsen SF. Factors affecting quality of life in patients with hidradenitis suppurativa. Arch Dermatol Res. (2020) 312:427–36. 10.1007/s00403-019-02025-5 31848682

[46] Fotiadou C Vakirlis E Ioannides D. Spotlight on adalimumab in the treatment of active moderate-to-severe hidradenitis suppurativa. Clin Cosmet Investig Dermatol. (2016) 9:367–72. 10.2147/CCID.S93619 27799806 PMC5076543

